# Cognitive Decline and Psychological Distress in Chronic Cardiovascular Patients: The Interplay of Mental Health, Cognition, and Disease Severity

**DOI:** 10.7759/cureus.77412

**Published:** 2025-01-14

**Authors:** Sher Bano, Zainab Salahuddin, Sameen Asim Choudhry, Muhammad Rumman Javed, Rabia Shakoor, Aisha Alyassi, Saliha Zahid, Maryum LNU, Muhammad Zohaib Amjad, Hammad Wasim

**Affiliations:** 1 Department of Psychology, Brain Wave Research Center, Islamabad, PAK; 2 Department of Clinical Psychology, Shifa Tameer-e-Millat University, Islamabad, PAK; 3 Department of Medicine, Nishtar Medical College, Bahawalpur, PAK; 4 Department of General Medicine, Great Western Hospital, Swindon, GBR; 5 Department of Medicine, Sheikh Shakhbout Medical City, Abu Dhabi, ARE; 6 Department of Medicine, Foundation University, Islamabad, PAK; 7 Department of Medicine, Tallaght University Hospital, Dublin, IRL; 8 Department of Medicine, Royal Berkshire Hospital, Reading, GBR; 9 Department of Medicine, Milton Keynes University Hospital, Milton Keynes, GBR

**Keywords:** cardiovascular diseases, cognitive impairment, perceived stress, psychological distress, quality of life

## Abstract

Aim

Cardiovascular diseases (CVD) are one of the leading causes of morbidity and mortality in the global population and cause notable psychosocial and psychological repercussions, including psychological distress and cognitive impairment. This research examines the correlation between psychological distress, cognitive impairment, and disease severity in patients with chronic CVD. The study seeks to establish interrelations between these factors and functional activity, quality of life, and general health by comparing specific demographic and clinical variables.

Method

This cross-sectional study was conducted among 100 chronic CVD patients aged 35 and above, recruited from cardiovascular care units and outpatient clinics. The sample included 43 (43%) males and 57 (57%) females. The Perceived Stress Scale was used to assess psychological distress, the Duke Activity Status Index for functional activity, the EQ-5D-5L for health-related quality of life, and the Mini-Mental State Examination for cognitive function. Data were analyzed using IBM SPSS Statistics software version 26 (IBM Corp., Armonk, NY, USA) to perform correlation studies on stress, cognition, activities, and quality of life and to compare these results between groups based on marital status, education level, employment status, and disease duration.

Results

The results revealed a significant positive relationship between stress and functional activity, r = 0.521, and quality of life, r = 0.698, indicating that moderate stress levels can positively impact activity levels and perceived quality of life. The level of stress was negatively related to cognition (r = -0.318, demonstrating the toxic impact of stress. Mentally compensated participants were stressed and showed weaker cognitive results than non-compensated individuals, especially those hospitalized. Comparing stress and mental performance by marital status, employment status, and education showed that education status affected stress and cognition in different ways, and widowed and retired individuals had higher stress levels and lower cognitive scores.

Conclusion

This study highlights the complex interplay between psychological distress, cognitive impairment, and disease severity in chronic CVD patients. Findings emphasize the need for integrated interventions that address psychological and mental health to improve overall patient care and health-related quality of life. These findings suggest the potential for developing targeted psychological and cognitive support interventions to enhance functional activity, reduce distress, and improve quality of life in chronic CVD patients. Tailored strategies for vulnerable groups, such as widowed and retired individuals, could help mitigate stress, preserve cognitive function, and enhance health outcomes in chronic disease settings.

## Introduction

CVDs remain the leading global cause of mortality, accounting for approximately 17.9 million deaths annually [1]. However, beyond the physical profile, CVDs are now considered to have significant psychological and cognitive effects. Most chronic cardiovascular disorders cause psychological symptoms such as anxiety and depression among patients with heart disease [2]. These patients often have coexisting cognitive dysfunction, which presents with symptoms including memory disorders, slowed executive processing, and compromised working memory; these factors make disease management more challenging and decrease HrQoL [3].

Recent research findings reveal that psychological distress positively interferes with cognitive function in CVD patients. Conversely, cognitive function has a similar positive impact on the level of psychological distress in the same patients. Stress-related factors may act as a catalyst to increase inflammation and compromise the integrity of the circuits within the brain that control cognition [4]. On the other hand, cognitive deterioration leads to a decline in self-care, thus raising psychological demands and reducing compliance with treatment [5]. Nonetheless, the relationship between mental health or cognition and disease severity in chronic cardiovascular patients is still not well illustrated. It is likely that disease duration, comorbid vital conditions, and medication regime compliance act as moderators in these associations [6].

CVDs, psychological distress, and cognitive decline highlight important clinical issues as well. Patients with higher psychological and cognitive impairment are at a higher risk of readmission to the hospital and overall mortality [7]. In addition, these comorbidities are costly, place a significant burden on healthcare systems, and require multidisciplinary care models that address both physical and mental health condition outcomes [8]. However, how these variables are interconnected and the consequences of their interconnection on the course of the disease also constitute a research concern.

This research aims to identify the complex interdependency between the deterioration of cognition, the presence of psychological symptoms, and the extent of the disease in chronic cardiovascular patients. In this cross-sectional study, we investigate the associations and correlates of these interconnected processes to enhance comprehensive patient care and favorable outcomes. In this context, this research aims to fill the gaps in understanding mediators and moderators in the interplay between various protective and risk factors relevant to this vulnerable group and lay the foundation for further advances in intervention development.

Rationale and objectives

CVDs encompass the most significant type of non-communicable disease that continues its increasing prevalence and impact on global healthcare. Although the bodily manifestations of these diseases cannot be doubted, their influence on a patient's mind or cognitive ability is one area that clinicians have not investigated adequately, despite its importance to the health of the human subject. Depression and cognitive impairment are among the common neuropsychiatric complaints seen in those with chronic CVD. However, patients presenting with these problems often remain undiagnosed, let alone receiving appropriate treatment. These conditions affect the patient's quality of life and lead to non-compliance with medical therapy, worsening of the disease, and detrimental clinical status.

Evaluations of cardiovascular health, cognition, and psychological distress are complicated by often coexisting vascular disease, neuroinflammation, and aluminium toxicity in context with existing chronic disease and related psychosocial factors. Nevertheless, while prior studies pay attention to these factors, they are usually studied independently, raising the question of how these associated factors interact within the same patient population. This creates a lack of systemic data, which confines strategic efforts for interventions that address these intertwining issues.

This study intends to fill this gap by determining the rates of mild cognitive impairment (MCI) and post-stroke depression (PSD) in chronic CVD patients and how disease severity moderates these two. The research is important for enhancing clinical practice knowledge by providing information on the prevention and management of these conditions. Additionally, the results will highlight the significance of using a coordinated approach to managing cardiovascular, cognitive, and psychological disorders to enhance patient care and the overall effectiveness of healthcare policies.

## Materials and methods

We investigated the connection between cognitive impairment, psychological symptoms, and disease complexity in 100 chronic cardiovascular patients (N = 100, 43% males, 57% females) aged 35 and above. These patients were recruited from cardiovascular care units and outpatient clinics. Participants were required to be aged 35 years or above, suffering from at least one form of chronic cardiovascular disease, and willing to sign a consent form. The inclusion criterion was being over 35 years of age and suffering from at least one form of chronic cardiovascular disease. Exclusion criteria included prior neurological diagnosis, psychiatric disorders not related to cardiovascular health, inability to provide informed consent, or lack of current involvement in active clinical management. Data collection occurred from September 2024 to November 2024 through structured questionnaires. The sample size was estimated using the WHO sample size calculator at a 95% confidence level with a prevalence of 0.8. However, due to time and resource constraints, this study was carried out with 100 participants (N = 100, 100%), selected purposively, and recruited to include diverse cardiovascular diseases, severity, and ages.

The Duke Activity Status Index (DASI), developed by Hlatky MA et al., 1987 (Appendix 1), assessed patients' functional capacity and physical activity levels. This scale consists of 12 items measuring physical activity levels [9]. The EQ-5D-5L, developed by Stolk E et al., 2011 (Appendix 2), was utilized to evaluate health-related quality of life across five dimensions: mobility, self-care, usual activities, pain/discomfort, and anxiety/depression [10]. The Perceived Stress Scale (PSS), developed by Cohen S et al. in 1983 (Appendix 3), was used to measure the degree of stress perceived by participants over the past month [11]. Cognitive function was assessed using the Mini-Mental State Examination (MMSE), developed by Folstein MF et al. in 1975 (Appendix 4), which consists of 30 items evaluating cognitive domains such as orientation, memory, attention, and language [12]. All instruments used in this study were validated through reliability analyses, including Cronbach's alpha, to ensure acceptable reliability levels. Participants' responses were recorded on Likert scales adapted for each specific instrument during data collection.

The data were analyzed using IBM SPSS Statistics, Version 26 (IBM Corp., Armonk, NY, USA). Ethical approval for the study was obtained from the Institutional Review Board (IRB-2024-0025) of the Brain Wave and Research Center, Islamabad, which operates as an independent research organization with its own review board (Appendix 5). Participants were informed about the purpose of the study, and their privacy, confidentiality, and anonymity were maintained throughout the research. The collected data were used solely for academic purposes, and participants' autonomy and self-esteem were respected during all stages of the study.

## Results

Table 1 shows the mean age was 60 (SD = 15) years. Out of 350, the sample included 43% males and 57% females. Regarding education level, 10% had a high school diploma, 35% had a college degree, 33% had a master's degree, and the remaining 22% had a doctorate. The participants' employment status was another area of consensus; 34% were employed, 34% were unemployed, and the other 32% were retired. Regarding marital status, about slightly more than half, 55% of the participants were married, while the others were widowed (25%), divorced (18%), and single (2%). Regarding cardiovascular diagnosis time, 24% had a cardiovascular diagnosis for 4-8 months, 41% for one year, and 35% for over one year. Also, 70 percent of the individuals had been admitted to the hospital for CVD, with thirty percent not having such an experience.

**Table 1:** Demographic characteristics of participants (N=100). F: Frequency; %: Percentage; CVD: Cardiovascular diseases. Mean age = 60 years (SD = 15).

Variable	F	%
Male	43	43
Female	57	57
Education	-	-
High School	10	10
Bachelor’s degree	35	35
Master’s degree	33	33
Doctorate	22	22
Employment	-	-
Employed	34	34
Unemployed	34	34
Retired	32	32
Marital Status	-	-
Single	2	2
Married	55	55
Divorced	18	18
Widowed	25	25
Duration of Cardiovascular Diagnosis	-	-
4-8 Months	24	24
1 Year	41	41
More than 1 Year	35	35
Hospitalization due to Cardiovascular Diseases	-	-
Yes	70	70
No	30	30

As presented in Table 2, the correlation coefficients of the study variables are presented. Positive and significant correlations were observed between PSS and DASI (r = 0.521, p < 0.01) and PSS and EQ-5D (r = 0.698, p < 0.01), indicating that high stress levels are statistically associated with great functional ability and better quality of life score. However, negative correlations were observed between MMSE and PSS (r = -0.318, p < 0.01), DASI (r = -0.451, p < 0.01), and EQ-5D (r = -0.393, p < 0.01), suggesting that higher stress, activity levels, and quality-of-life scores are linked to lower cognitive performance.

**Table 2:** Intercorrelations between study variables. *p < 0.05; **p < 0.01; ***p < 0.001. Values represent Pearson’s correlation coefficients (r). Positive correlations indicate direct relationships, while negative correlations indicate inverse relationships.

Variable	Perceived Stress	Duke Activity Status	Health-related Quality of Life States	Mental State
Perceived Stress Scale	-	-	-	-
Duke Activity Status Index	0.521^**^	-	-	-
Health-related quality of life states	0.698^**^	0.524^**^	-	-
Mini-Mental State Examination	-0.318^**^	-0.451^**^	-0.393^**^	-

Table 3 presents the characteristics of respondents based on hospitalization status for cardiovascular diseases. The mean PSS scores were significantly higher among participants who had been hospitalized (M = 40.8, SD = 6.9) compared to non-hospitalized participants (M = 33.2, SD = 3.7) with a large effect size (Cohen’s d = 1.37). Hospitalized participants reported significantly higher DASI scores (M = 18.3, SD = 4.4) than non-hospitalized participants (M = 14.2, SD = 2.8), with a large effect size (Cohen’s d = 1.11). For quality of life, a higher score was rated on the EQ-5D among the hospitalized participants (M = 16.6, SD = 3.9) as compared to non-hospitalized participants (M = 12.5, SD =3.2), large effect size (Cohen’s d = 1.15). However, the non-hospitalized patients achieved higher mean scores of the MMSE (M = 27.7, SD =1.9) compared to both the hospitalized patients (M = 24.9, SD = 5.8) with small effect sizes (Cohen’s d = 0.65). All differences were found statistically significant (p < 0.05), with p-values for some variables reaching < 0.001.

**Table 3:** Comparison among variables (hospitalization due to cardiovascular diseases). M = mean; SD = standard deviation; LL = lower limit; UL = upper limit; CI = confidence interval; ES = effect size; PSS = Perceived Stress Scale; DASI = Duke Activity Status Index; EQ-5D = EuroQol-5 Dimension; MMSE = Mini-Mental State Examination. *p < 0.05; **p < 0.01; ***p < 0.001. All differences were analyzed using independent t-tests. The effect size (Cohen's d) interpretation was small (d = 0.2), medium (d = 0.5), and large (d ≥ 0.8).

Variable	Yes		No		t	P	CI 95%		Cohen’s D
-	M	SD	M	SD	-	-	LL	UL	-
Perceived Stress Scale	40.8	6.9	33.2	3.7	5.685	0.000	4.9	10.2	1.37
Duke Activity Status Index	18.3	4.4	14.2	2.8	4.685	0.000	2.4	5.8	1.11
EQ-5D-5L	16.6	3.9	12.5	3.2	5.063	0.062	2.5	5.6	1.15
Mini-Mental State Examination	24.9	5.8	27.7	1.9	-2.532	0.000	-4.9	-.59	0.65

Table 4 compares variables across marital statuses: single, married, divorced, and widowed. Widowed participants had a significantly higher PSS score (M = 43.4, SD = 7.1) than the other groups: divorced (M = 40.4, SD = 7.1), married (M = 35.9, SD = 5.5) and single (M = 35.0, SD = 8.5) with large effect size (η² = 0.222, where η² ≥ 0.14 is considered significant). For the Duke Activity Status Index (DASI), widowed participants had the highest physical activity levels (M = 21.6, SD = 3.5), with a large effect size (η² = 0.388). Widowed participants had the highest score on the EQ-5D-5L quality of life index (M = 18.6, SD = 2.7) and a medium effect size (η² = 0.288). Single participants scored the highest on the MMSE (M = 29.0, SD = 1.4), followed by married (M = 27.4, SD = 2.7), divorced (M = 25.6, SD = 5.4), and widowed participants (M = 21.9, SD = 6.9), with a small effect size (η² = 0.203).

**Table 4:** Comparison of variables (marital status). M = mean; F = F-ratio; η² = effect size. ***p < 0.001; **p < 0.01; *p < 0.05.

Variable	Single	Married	Divorced	Widowed	F (3,96)	η2
-	M±S.D	M±S.D	M±S.D	M±S.D	-	-
Perceived Stress	35.0±8.5	35.9±5.5	40.4±7.1	43.4±7.1	9.1^***^	0.222
Duke Activity Status	15.5±0.7	15.1±3.4	16.9±4.1	21.6±3.5	20.3^***^	0.388
EQ-5D-5L	12.5±2.1	13.6±3.7	16.7±3.8	18.6±2.7	12.9^***^	0.288
Mental State	29.0± 1.4	27.4±2.7	25.6±5.4	21.9±6.9	8.2^***^	0.203

Table 5 highlights differences in key variables across three employment statuses: employed, unemployed, and retired. The Perceived Stress Scale (PSS) results indicate that retired participants had higher stress levels (M = 42.8, SD = 6.2) compared to unemployed (M = 37.1, SD = 7.3) and employed participants (M = 36.0, SD = 5.6) with a moderate effect size η² = 0.182, where 0.06 ≤ η² 0.14 is moderate). Retired participants had significantly higher DASI scores (M = 20.8; SD = 3.7) than unemployed (M = 16.3; SD = 3.7), t (77) = 2.12, p = 0.035) and employed participants (M = 14.3; SD = 3.1, t (77) = 5.59, p < 0.001), with a large effect size (η² = 0.381). For quality of life, retired participants showed the highest EQ-5D-5L scores (M = 17.6, SD = 3.6), followed by unemployed participants (M = 15.6, SD = 3.9). Employed participants had the lowest scores (M = 13.1, SD = 3.4) with a moderate effect size (η² = 0.203). Using the MMSE, employed participants scored the highest for cognitive performance (M = 27.4, SD = 2.2), while the unemployed (M = 25.4, SD = 5.5) and retired participants had (M = 24.3, SD = 6.4), with a moderate effect size (η² = 0.201).

**Table 5:** Comparison of variables (employment). M = mean; F = F-ratio; η² = effect size. ***p < 0.001; **p < 0.01; *p < 0.05.

Variable	Employed	Unemployed	Retired	F (2,97)	η2
-	M±S.D	M±S.D	M±S.D	-	-
Perceived Stress Scale	36.0±5.6	37.1±7.3	42.8±6.2	10.8^***^	0.182
Duke Activity Status Index	14.3±3.1	16.3±3.7	20.8±3.7	29.9^***^	0.381
EQ-5D-5L	13.1±3.4	15.6±3.9	17.6±3.6	12.3^***^	0.203
Mini-Mental State Examination	27.4± 2.2	25.4±5.5	24.3±6.4	3.1^***^	0.201

Table 6 compares variables across education levels: high school, bachelor's, master's, and doctorate. Doctoral-level participants were more stressed (M = 42.1, SD = 5.7) than master's level participants (M = 40.6, SD = 7.9), with a moderate effect size (η² = 0.184). Regarding physical activity, respondents with master's degrees had the highest DASI scores (M = 18.8, SD = 4.4), followed by those with high school education (M = 18.1, SD = 4.0). The effect size was moderate (η² = 0.184). Masters holders recorded the highest EQ-5D-5L index mean score on quality of life (M = 17.5, SD = 3.5), while those with a bachelor’s degree had the lowest score (M = 13.6, SD = 3.7)). The effect size was moderate, amounting to η² = 0.180. For cognitive performance (MMSE), participants with bachelor's degrees scored the highest (M = 27.2, SD = 2.3), slightly outperforming other groups. However, the effect size was small (η² = 0.081).

**Table 6:** Comparison of variables (education). M = mean; F = F-ratio; η² = effect size. ***p < 0.001; **p < 0.01; *p < 0.05.

Variable	High School	Bachelor’s Degree	Master’s Degree	Doctorate Degree	F (3,96)	η2
-	M±S.D	M±S.D	M±S.D	M±S.D	-	-
Perceived Stress Scale	35.4±5.7	35.3±5.4	40.6±7.9	42.1±5.7	7.2^***^	0.184
Duke Activity Status Index	18.1±4.0	14.8±3.3	18.8±4.4	17.5±4.9	5.7^***^	0.151
EQ-5D-5L	13.7±4.1	13.6±3.7	17.5±3.5	15.7±4.1	7.0^***^	0.180
Mini-Mental State Examination	26.3± 3.6	27.2±2.3	23.8±6.6	26.1±5.7	2.8^**^	0.081

Table 7 shows the comparison based on the duration of cardiovascular diagnosis: 4-8 months, 1 year, and more than 1 year. Regarding the Perceived Stress Scale (PSS), no significant differences were found between the groups, though stress levels were slightly higher for those diagnosed for more than 1 year (M = 39.4, SD = 7.3), with a moderate effect size (η² = 0.184). The patients with a diagnosis for more than a year had a much higher score in terms of their perceived activity level using DASI (M = 18.8, SD = 4.3) than those who had been diagnosed between 4 to 8 months (M = 14.8, SD = 3.6) or 1 year (M = 16.9, SD = 4.4). Again, the effect size was small (η² = 0.151). EQ-5D-5L findings also revealed that no statistically significant differences were found between groups in the study concerning quality of life with a small effect (η² = 0.180). In cognitive performance assessed using the MMSE, participants diagnosed for over 1 year showed lower scores (M = 22.8, SD = 5.7) than those diagnosed for 4-8 months (M = 27.1, SD = 4.6) or 1 year (M = 27.4, SD = 3.6). The effect size for MMSE was small (η² = 0.081).

**Table 7:** Comparison of variables (duration of cardiovascular diagnosis). M = mean; F = F-ratio; η² = effect size. ***p < 0.001; **p < 0.01; *p < 0.05.

Variable	4-8 Months	1 Year	More than 1 Year	F (2,97)	η2
-	M±S.D	M±S.D	M±S.D	-	-
Perceived Stress Scale	37.4±6.8	38.5±6.9	39.4±7.3	0.6	0.184
Duke Activity Status Index	14.8±3.6	16.9±4.4	18.8±4.3	6.9^***^	0.151
EQ-5D-5L	15.1±3.9	15.0±4.2	15.9±4.1	0.6	0.180
Mini-Mental State Examination	27.1± 4.6	27.4±3.6	22.8±5.7	10.4^***^	0.081

Table 8 lists the demographic characteristics of respondents, including gender, employment, marital status, and hospitalization. This shows a significant difference in gender and employment status (p = 0.005, χ² (2) = 10.6). Regarding employment status, most of the males were either working (19 males, 44.2%) or retired (17 males, 39.5%); the remaining were unemployed (7 males, 16.3%). Among the females, 27 (47.4%) were unemployed, 15 (26.3%) were employed, and 15 (26.3%) were retired. However, there was no statistically significant association between gender and marital status (p = 0.374, χ²(3) = 3.1). Most males were married (27 males, 62.8%), 10 (23.3%) were widowed, and 6 (14.0%) were divorced. However, most female respondents were married (28 females, 49.1%), while 15 (26.3%) were widowed, 12 (21.1%) were divorced, and only 2 (3.5%) were single. Employment status and hospitalization status were significant (p = 0.000, χ²(2) = 22.7), and marital status was also significant with the hospitalization status (p = 0.000, χ²(3) = 22.6). Regarding hospitalized individuals, 30 (42.9%) were retired, 26 (37.1%) were unemployed, and 14 (20.0%) were employed. If not hospitalized, 20 (66.7%) were working; the rest were unemployed (8, 26.7%) or retired (2, 6.7%). For marital status, 28 (40.0%) of hospitalized patients were married, 25 (35.7%) were widowed, 15 (21.4%) were divorced, and only 2 (2.9%) were single. In non-hospitalized participants, 27 (90.0%) were married, while 3 (10.0%) were divorced, with no widowed or single participants.

**Table 8:** Descriptive statistics of demographic variables. F = frequency; % = percentage; χ² = chi-square value; p = significance level. ***p < 0.001; *p < 0.01. Percentages were calculated using the chi-square test with significance set at p < 0.05.

Variables	f	Employed	Un-employed	Retired	p	x^2^	Single	Married	Divorce	Widow	p	x^2^
Gender	-	-	-	-	-	-	-	-	-	-	-	-
Male	43	19 (44.2%)	7 (16.3%)	17 (39.5%)	0.005*	10.6	0	27 (62.8%)	6 (14.0%)	10 (23.3%)	0.374	3.1
Female	57	15 (26.3%)	27 (47.4%)	15 (26.3%)	-	-	2 (3.5%)	28 (49.1%)	12 (21.1%)	15 (26.3%)	-	-
Hospitalization	-	-	-	-	-	-	-	-	-	-	-	-
Yes	70	14 (20.0%)	26 (37.1%)	30 (42.9%)	0.000***	22.7	2 (2.9%)	28 (40.0%)	15 (21.4%)	25 (35.7%)	0.000	22.6
No	30	20 (66.7%)	8 (26.7%)	2 (6.7%)	-	-	0	27 (90.0%)	3 (10.0%)	0	-	-

## Discussion

Chronic cognitive impairment and psychological disorders have been explored, highlighting the chronological interconnection between cardiovascular patients. This study generates new knowledge by replicating prior findings regarding the link between stress and activity levels, coupled with cognitive function and quality of life, and examining these relationships within different demographic and clinical categories.

The positive and mild relationship between PSS and DASI shows that highly stressed individuals might also exhibit high functional capacity. This relationship aligns with the stress-related activation theory proposed by [13], which asserts that stress, as long as it is not chronic or disabling, can energize physical activity in some groups. Likewise, PSS more modestly correlates positively with EQ-5D-5L, which may mean that stress can be accompanied by perceived enhanced quality of life, where stress may be related to engaging role demands and the fuzzy interconnection between high function and mental health [14].

However, the negative association coefficients of the Mini-Mental State Examination (MMSE) with PSS, DASI, and EQ-5D-5L suggest that increased stress and activity levels may impair cognition. These results are in concordance with existing studies that show stress, especially chronic stress, adversely impacts cognitive function, particularly in old age [15].

The results also showed significant differences when comparing the hospitalized and non-hospitalized participants. The total stress level was found to be significantly higher in hospitalized individuals as measured by PSS; this is expected because admission to the hospital comes with a certain amount of psychological load [16]. Higher DASI scores obtained from the admitted patients may be attributed to higher functional activity levels before cardiovascular events, consistent with a hypothesis that increased activity might lead to such episodes [17]. Contrary to expectations, subjects in the hospital had slightly improved EQ-5D-5L scores, perhaps due to the psychological and functional advantages organized medical care offers during a disease flare-up, as suggested by [18]. On the other hand, the lowered MMSE scores among hospitalized patients pointed to the possible direct cost of severe cardiovascular diseases on cognitive function [19].

The results for marital status are equally important. Married participants had significantly lower means for PSS and DASI than the other marital status categories; widowed participants had the highest means for PSS and DASI; divorced and separated participants had intermediate means for both PSS and DASI, with single participants close behind the divorced and separated participants when ranked by DASI score but with a slightly higher mean PSS score [20]. Higher EQ-5D-5L scores observed in this group add to the evidence for protective factors that would buffer the consequences of stress on quality of life. However, widowed participants also had the lowest MMSE scores, which pointed to a cognition problem faced by participants experiencing stress and bereavement in chronic conditions.

The results also highlighted employment status as an important predictor of health status. Retirement-age participants had the highest PSS, probably due to the financial and other social changes accompanying most retirees' experiences. Still, thanks to a higher DASI and EQ-5D-5L, they pay much attention to health and quality of life during this period. Nevertheless, the relatively higher MMSE scores we found in employed participants are consistent with the notion that paid work benefits cognition [21]. These results raise awareness that employment, while providing cognitive benefits, might also help attenuate or moderate perceived stress levels.

The education level also differentiates the dynamics of psychological and cognitive effects. Although education at a higher level brought about higher Perceived Stress Scale (PSS) scores, it improved self-reported physical activity and quality of life (DASI and EQ-5D-5L). This push-pull effect paradoxes the roles and stress-mediating capabilities related to higher education [13]. However, the slightly enhanced executive cognitive ability in participants with a bachelor's degree implies that education as a protective factor for cognition may reach a tipping point beyond which it does not further improve.

Cardiovascular diagnosis duration was neither beneficial nor detrimental. However, there were no significant differences in stress (PSS) and quality of life (EQ-5D-5L) between groups. At the same time, a prolonged diagnosis was strongly associated with better activity (DASI), probably due to adaptation to the disability. However, we would like to stress that the noted lower MMSE scores in participants with prolonged diagnostics indicate the stereotyped rate of chronic impact of cardiovascular diseases on cognition [19].

Employment status was another significant factor that had different effects on gender; men more often represented the employed categories, and women were more often unemployed. Gender disparities highlighted here conform with other employment outcomes trends and the relation of these trends to health and economic stability [21]. Also, the result showing that retired and unemployed participants had significantly higher hospitalization rates suggests that employment could be a protective factor in preventing worse illness experiences, mainly attributable to higher healthcare utilization among the employed participants [17].

However, some constraints can be identified in this research study as follows. First, due to the cross-sectional design of the work, causal relationships between psychological distress, cognitive dysfunction, and disease severity are impossible to determine. Secondly, the sample size of 100 is quite informative. However, it may not be sufficient to capture the heterogeneity of this population of chronic cardiovascular patients, at least in terms of demographic and clinical characteristics. Additionally, the present work did not distinguish the effects of stress on cognitive function according to medication schedules, the presence of comorbidities, or lifestyle practices that might affect stress, cognition, and quality of life. Another limitation is the use of self-reporting instruments, which can produce bias by reflecting participants' subjective experiences. Finally, this study was limited to short-term evaluations; therefore, more investigation should be done to explore chronic stress and cognition in patients with coronary heart disease over the long term.

## Conclusions

The relationship between psychological distress, cognitive status, and disease severity has been demonstrated in this study among chronic cardiovascular patients. The results indicate that moderate stress enhances functional activity and perceived quality of life, while chronic stress impairs cognition. The study also highlights the need for the development of specific stress intervention strategies for high-risk subgroups in the elderly population, including the widowed and the retired. Adopting psychosocial and cognitive treatments as additional strategies within cardiovascular care could advance treatment strategies and improve quality of life. Incorporating stress management techniques, such as cognitive-behavioral therapy and mindfulness-based interventions, could help mitigate psychological distress and preserve cognitive function. Additionally, regular cognitive training programs and social support initiatives may enhance mental resilience and overall well-being. Longitudinal studies could further clarify causal links between stress, cognition, and disease progression, providing a stronger foundation for tailored clinical practices. It may also slow the advancement of chronic conditions. Further research with larger and more heterogeneous samples, and research employing longitudinal designs, is suggested to corroborate these findings and guide more specific interventional efforts.

## Figures and Tables

**Figure 1:**
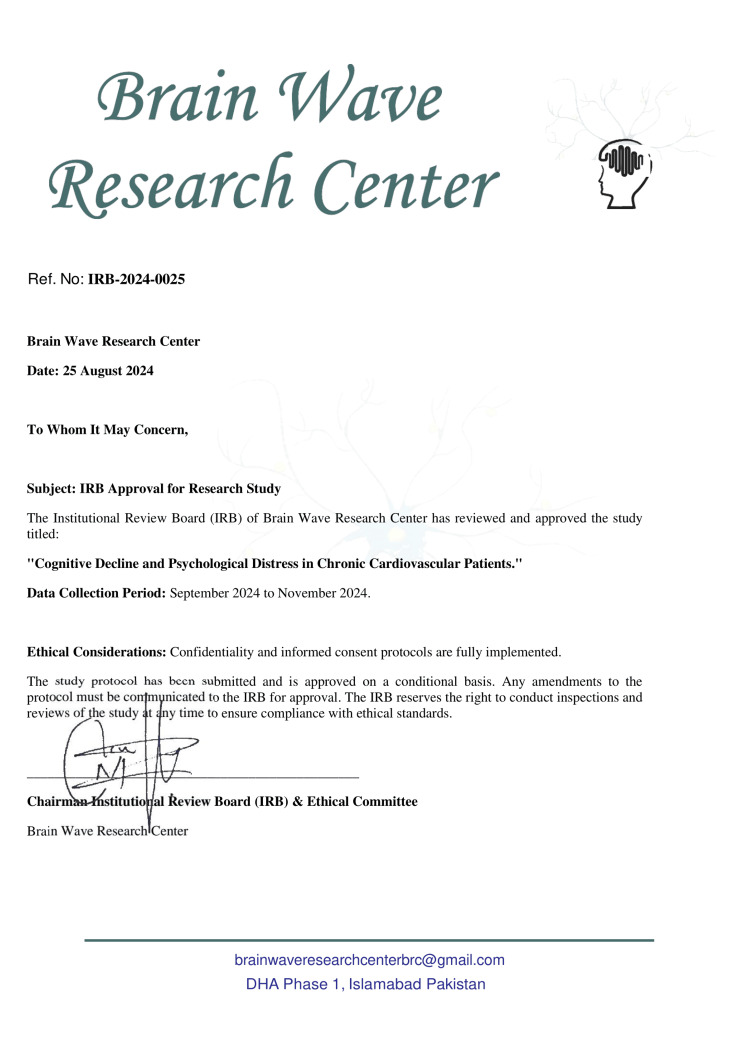
IRB approval.

## Appendices

Appendix 1 

**Table 9:** Duke Activity Status Index. Source: Hlatky MA et al., 1987 [9].

Item	Response
Can you take care of yourself (eating, dressing, bathing, or using the toilet?)	-
Can you walk indoors, such as around your house?	-
Can you walk a block or two on level ground?	-
Can you climb a flight of stairs or walk up a hill?	-
Can you run a short distance?	-
Can you do light work around the house, like dusting or washing dishes?	-
Can you do moderate work around the house, like vacuuming, sweeping floors, or carrying groceries?	-
Can you do heavy work around the house, like scrubbing floors or lifting or moving heavy furniture?	-
Can you do yard work like raking leaves, weeding, or pushing a power mower?	-
Can you have sexual relations?	-
Can you participate in moderate recreational activities like golf, bowling, dancing, doubles tennis, or throwing a baseball or football?	-
Can you participate in strenuous sports like swimming, singles tennis, football, basketball, or skiing?	-

Appendix 2 

**Table 10:** EQ-5D-5L. Source: Stolk E et al., 2011 [10].

Item	Response
MOBILITY I have no problems in walking about I have slight problems in walking about I have moderate problems in walking about I have severe problems in walking about I am unable to walk about	-
SELF-CARE I have no problems washing or dressing myself I have slight problems washing or dressing myself I have moderate problems washing or dressing myself I have severe problems washing or dressing myself I am unable to wash or dress myself	-
USUAL ACTIVITIES (e.g. work, study, housework, family or leisure activities) I have no problems doing my usual activities I have slight problems doing my usual activities I have moderate problems doing my usual activities I have severe problems doing my usual activities I am unable to do my usual activities	-
PAIN / DISCOMFORT I have no pain or discomfort I have slight pain or discomfort I have moderate pain or discomfort I have severe pain or discomfort I have extreme pain or discomfort	-
ANXIETY / DEPRESSION I am not anxious or depressed I am slightly anxious or depressed I am moderately anxious or depressed I am severely anxious or depressed I am extremely anxious or depressed	-

Appendix 3 

**Table 11:** Perceived Stress Scale. Source: Cohen S et al., 1983 [11].

Item	Response
In the last month, how often have you been upset because of something that happened unexpectedly?	-
In the last month, how often have you felt that you were unable to control the important things in your life?	-
In the last month, how often have you felt nervous and stressed?	-
In the last month, how often have you felt confident about handling your personal problems?	-
In the last month, how often have you felt that things were going your way?	-
In the last month, how often have you found that you could not cope with everything you had to do?	-
In the last month, how often have you been able to control irritations in your life?	-
In the last month, how often have you felt that you were on top of things?	-
In the last month, how often have you been angered because of things that happened that were outside of your control?	-
In the last month, how often have you felt difficulties were piling up so high that you could not overcome them?	-

Appendix 4 

**Table 12:** Mini mental state exam. Source: Folstein MF et al., 1975 [12].

Section	Task	Points
Orientation	What is the (year) (season) (date) (day) (month)?	5
-	Where are we (state) (country) (town) (hospital) (floor)?	5
Registration	Name 3 objects: 1 second to say each. Then ask the patient all 3 after you have said them. Give 1 point for each correct answer. Then repeat them until he/she learns all 3. Count trials and record. Trials	3
Attention and Calculation	Serial 7's. 1 point for each correct answer. Stop after 5 answers. Alternatively, spell "world" backward	5
Recall	Ask for the 3 objects repeated above. Give 1 point for each correct answer	3
Language	Name a pencil and watch	2
-	Repeat the following “No ifs and buts"	1
-	Follow a 3-stage command: “Take a paper in your hand fold it in half and put it on the floor”	3
-	Read and obey the following: CLOSE YOUR EYES	1
-	Write a sentence	1
-	Copy the design shown	1
Total	-	30

Appendix 5 
